# Secreted enzyme uptake masks the *in vivo* phenotype of macrophage-specific lysosomal acid lipase deletion

**DOI:** 10.1016/j.molmet.2026.102369

**Published:** 2026-04-16

**Authors:** Suravi Mukherjee, Melanie Korbelius, Anita Pirchheim, Birgit Schwarz, Malena Diaz, Laszlo Schooltink, Gernot F. Grabner, Nemanja Vujić, Dagmar Kratky

**Affiliations:** 1 Department of Molecular Biology and Biochemistry, Gottfried Schatz Research Center, Molecular Biology and Biochemistry, Medical University of Graz, Graz, Austria; 2 BioTechMed-Graz, Graz, Austria

**Keywords:** LAL, LAL deficiency, LAL-Deficient mouse, LAL secretion, LAL uptake, Cholesteryl ester storage disease, Wolman disease

## Abstract

**Background and hypothesis:**

Lysosomal acid lipase (LAL) is so far the only known intracellular enzyme that is capable of hydrolyzing triglycerides and cholesteryl esters at an acidic pH inside the lysosome. Mutations in the LAL-encoding Lipa gene cause a rare autosomal recessive lysosomal storage disorder in humans with massive lipid accumulation. In mice, the loss of systemic LAL is associated with severe lipid accumulation, particularly in the liver and small intestine, accompanied by infiltration of lipid-filled CD68+-TREM2+ macrophages. We hypothesize that macrophages are among the key players in LAL deficiency and are responsible for lipid accumulation in the affected tissues.

**Methods:**

We generated macrophage (mac)- and macrophage/enterocyte-specific (mac/int-) LAL KO mice and performed morphological, histopathological, and functional analyses under chow- and high-fat/high-cholesterol diet-fed conditions.

**Results:**

We observed that neither macLAL-KO nor mac/int-LAL KO mice replicated the phenotype of whole-body LAL KO mice, as lipoprotein secretion, lipid absorption, and lipid accumulation remained unaffected. However, the absence of macrophage LAL ameliorated diet-induced obesity in both mouse lines. Notably, the lipid accumulation observed in the lysosomes of macrophages from whole-body LAL KO mice was absent in macrophages from macLAL-KO mice, attributable to residual LAL enzyme activity despite genetic ablation. Treatment of macrophages from whole-body LAL KO mice with conditioned medium of hepatocytes from macLAL-KO mice effectively prevented lipid accumulation.

**Conclusion:**

These findings suggest that LAL secreted from hepatocytes, macrophages, and possibly other cell types in vivo corrects the phenotype of cell type-specific LAL deficiency, a key insight for guiding future gene therapy strategies.

## Highligths

• Macrophage LAL deficiency fails to mimic whole-body LAL knockout phenotype.
• Residual LAL activity prevents lipid buildup in macLAL-KO macrophages.
• Hepatocyte-derived LAL prevents lipid accumulation in LAL KO macrophages.
• Hepatocyte-secreted LAL rescues macrophage KO phenotype.
• LAL uptake by LAL KO macrophages is key for correcting tissue lipid overload.

## Graphical abstract

**Figure:**
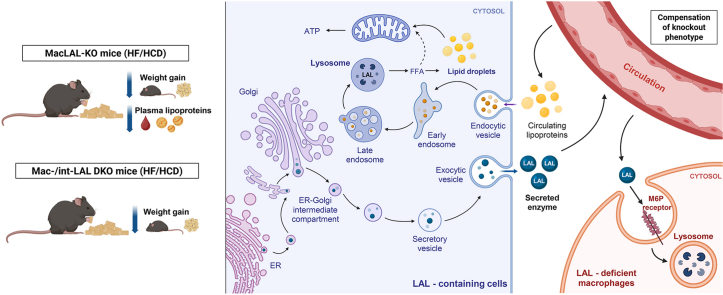

## 1 Introduction

Cholesterol homeostasis is vital for membrane fluidity, endocrine signaling, and intracellular trafficking [1]. Dietary cholesterol and triglycerides (TG) are absorbed by enterocytes, packaged into chylomicrons, and released via the lymphatic system. In the circulation, TG are hydrolyzed by lipoprotein lipase (LPL) to generate chylomicron remnants that are taken up by hepatocytes. Upon fasting, the liver secretes very low-density lipoproteins (VLDL), which are converted to low-density lipoprotein (LDL) particles after LPL-mediated TG catabolism. LDL delivers cholesterol to extrahepatic cells through LDL receptor-mediated endocytosis [2,3]. In late endosomes/lysosomes, lipoprotein-derived cholesteryl esters (CE) get degraded by lysosomal acid lipase (LAL), releasing unesterified cholesterol that is exported by Niemann-Pick type C proteins to maintain cellular cholesterol balance [4,5]. To date, LAL is the only enzyme known to hydrolyze CE, TG, diacylglycerols, and retinyl esters in lysosomes (reviewed in [6]). LAL deficiency (LAL-D) in humans causes a rare autosomal recessive lysosomal storage disorder with two clinical manifestations of varying severity. Infants with early-onset LAL-D (formerly called Wolman disease) have ≤1% enzyme activity and no documented cases of survival beyond 12 months of age if patients are left untreated. They suffer from pronounced hepatosplenomegaly, adrenal calcification, incessant vomiting, and diarrhoea, leading to malabsorption, growth retardation, and ultimately liver failure [7,8]. In contrast, patients with late-onset LAL-D (formerly called CE storage disease) retain up to 10% enzymatic activity. As the symptoms are largely unspecific and overlap with other metabolic diseases associated with hypercholesterolemia [8,9], this may lead to a high rate of misdiagnosis.

Despite complete loss of LAL enzymatic activity, whole-body LAL knockout (KO) mice survive until adulthood, thus resembling late-onset LAL-D [10]. These mice accumulate CE and TG in various organs, including the spleen, liver, small intestine, and adrenal glands [11]. Adult LAL KO mice completely lack white adipose tissue (WAT) and therefore require constant energy supply [11,12]. In line, diminished VLDL secretion from the liver, improved glucose tolerance as well as lower plasma glucose levels indicate enhanced glucose utilization [12]. LAL KO mice are resistant to diet-induced obesity and have increased plasma LDL-TG and cholesterol when fed a western-type diet [13]. The most severely affected organs in these mice are the liver, in which massive CE accumulation can already be observed at the end of gestation, and the small intestine with increased CE and TG accumulation 4 weeks after birth [14].

Infiltrating macrophages are key players in tissue cholesterol clearance by taking up lipids and digesting them in lysosomes to release free cholesterol and fatty acids. They internalize LDL, VLDL, and modified lipoproteins through phagocytosis and scavenger receptor-mediated pathways (reviewed in [15]). The loss of LAL causes lysosomal lipid accumulation in macrophages, giving them a characteristic phenotype [12,16]. Clinically, these macrophages accumulate most prominently in the liver and the lamina propria of the small intestine (reviewed in [17]), a pattern similarly observed in whole-body LAL KO mice [11,13]. Intestinal lipid accumulation is mediated by a special subset of CD68^+^ macrophages with high expression of GPNMB and TREM2, referred to as lipid-associated macrophages (LAMs) [18,19]. Proteomics analysis also revealed the upregulation of the marker proteins for LAMs in LAL KO livers [20].

We hypothesized that infiltrating macrophages, together with hepatocytes, bear the greatest burden of LAL-D, driving ectopic lipid accumulation and chronic inflammation that ultimately results in (metabolic) dysfunction in the affected organs. To this end, we generated macrophage (mac)- and macrophage/enterocyte (mac/int)-specific LAL KO mouse models that enabled us to investigate the contribution of macrophages to the pathogenesis of LAL-D in the small intestine.

## 2 Materials and methods

### 2.1 Animals

Macrophage-specific LAL (macLAL) KO mice on the C57BL/6 J background were generated by crossing mice carrying a LoxP-flanked *Lipa* allele (Lipa-flox) with mice homozygously expressing Cre recombinase under the myeloid-specific LysM promoter. Corresponding Cre controls were used. The mac/int-LAL KO mice were generated by crossing Lipa-flox mice with homozygous LysM-Cre and enterocyte-specific Villin-Cre transgenic mice [18]. Floxed littermates lacking Cre served as controls.

All mice were maintained in a clean and temperature-controlled (22 °C ± 1 °C) environment with unlimited access to water and food on a regular 12-h light/dark cycle. Age- and sex-matched mice were fed either chow diet (4.1% crude fat; Altromin 1324, Lage, Germany) or challenged with a high-fat/high-cholesterol diet (HF/HCD; 34.6% crude fat, 1% cholesterol; Ssniff®, Soest, Germany) for 12 weeks at 10–12 weeks of age. In selected experiments, WT or LAL KO mice [21,22] were included as a phenotype control. Unless stated otherwise, the mice were fasted for 12 h prior to sacrifice by cervical dislocation. For non-terminal *in vivo* assays, mice were fed chow diet until 12 weeks of age; the same mice were then subjected to HF/HCD, reassessed *in vivo* after the feeding intervention, and euthanized. Tissues for post-mortem assays were obtained from an independent cohort maintained on a chow diet until 24 weeks of age.

All animal experiments were performed according to the European Directive 2010/63/EU in compliance with national laws and approved by the Austrian Federal Ministry of Education, Science and Research, Vienna, Austria (BMWFW-66.010/0109-WF/V/3b/2015, BMWFW-66.010/0082-WF/V/3b/2017, 2022–0.920.281, 2024–0.518.941).

### 2.2 Energy metabolism *in vivo*

Chow- and HF/HCD-fed macLAL-KO, mac/int-LAL KO, and corresponding control mice were single-housed in metabolic cages (Phenomaster; TSE Systems, Bad Homburg, Germany) for 3–4 days to measure food and water intake. Energy expenditure was assessed by indirect calorimetry in 15-min intervals.

### 2.3 Lipid parameters in plasma and tissues

After 12 h of fasting, blood was collected into EDTA-containing vials through puncture of the *vena facialis* and centrifuged for 7 min at 5,204×*g* at 4 °C to isolate plasma. The concentrations of circulating TG and total cholesterol (TC) (DiaSys, Holzheim, Germany) were measured spectrophotometrically using enzymatic kits according to the manufacturers’ instructions.

The lipid content of the liver and small intestine was quantitated as described previously with minor modifications [23]. Briefly, lipids from the tissue lysates were extracted using 6 ml of chloroform:methanol (v:v; 2:1) for 2 h at room temperature (RT). After centrifugation at 3,113×*g* for 15 min, the organic phase was collected, mixed with 1.2 ml PBS by vortexing, and centrifuged at 778×*g* for 15 min. The organic phase was again collected, 200 μl of 2% Triton-X-100 in chloroform was added, and the samples were dried under a stream of nitrogen. The extracted lipids were resuspended in 200 μl ddH_2_O and measured using the above-mentioned enzymatic kits. All values were normalized to protein concentrations.

### 2.4 Fast protein liquid chromatography (FPLC)

Plasma was obtained from mice fasted for 12 h as previously described. A pool of 200 μl plasma per genotype was run on the ÄKTA pure™ chromatography system (Cytiva, Marlborough, MA) equipped with a Superose 6 column (Amersham Biosciences, Piscataway, NJ). The lipoproteins were eluted with 10 mM Tris–HCl, 1 mM EDTA, 0.9% NaCl, and 0.02% NaN_3_ (pH 7.4). Fractions of 0.5 ml each were collected and TG and TC concentrations were determined enzymatically using above mentioned kits. To enhance sensitivity, the reagents were supplemented with sodium 3,5-dichloro-2-hydroxy-benzenesulfonate.

### 2.5 VLDL secretion assay

The mice were fasted for 16 h and then i.p. injected with tyloxapol (500 mg/kg body weight) to inhibit peripheral lipolysis. Blood was collected into EDTA-containing vials before and 2, 4, and 6 h after injection. Plasma was isolated by centrifugation for 10 min at 1,301×*g* at 22 °C and TG concentrations were determined as described above.

### 2.6 Chylomicron secretion assay

The mice were fasted for 4 h and then i.p. injected with tyloxapol (500 mg/kg body weight) to inhibit peripheral lipolysis. Thirty minutes after the injection, the mice were gavaged with 200 μl olive oil (for mice fed with normal chow diet) or olive oil containing 1% cholesterol (for mice fed with HF/HCD). Blood was collected into EDTA-containing vials before and 2, 4, and 6 h after gavage, plasma was isolated, and TG concentrations were determined as described above.

### 2.7 Gut transit time

After 12 h of fasting, the mice were gavaged with 200 μl Evans blue suspension (5% Evans blue (Sigma Aldrich, St. Louis, MI) and 5% gum Arabic (Roth, Karlsruhe, Germany)) in PBS. The mice were then given free access to water, and the time until Evans blue was detected in their feces was recorded.

### 2.8 Fecal lipid content estimation

Fecal pellets were collected for about 7 days from HF/HCD-fed, single-housed control and mac/int-LAL KO mice. The feces were lyophilized and pulverized using mortar and pestle. Lipids from 20 mg of feces were extracted with an 80-fold excess of a hexane:isopropanol (3:2) solution by rotating for 2 h at RT. The extracts were then centrifuged at 3,113×*g* for 15 min at 4 °C and the organic phase was decanted into a new vial. To the extracts, 100 μl of 2% Triton X-100 in chloroform was added and the samples were vortexed and dried under a stream of nitrogen. Finally, the samples were redissolved in 200 μl ddH_2_O and TG and TC concentrations were measured as described above. Free cholesterol (FC) was also measured enzymatically (mti Diagnostics GmbH, Idstein, Germany) and CE concentrations were calculated by subtracting FC from TC levels.

### 2.9 Oil red O (ORO) staining

Livers and small intestine segments (duodenum, jejunum, ileum) were isolated from mice fed chow or HF/HCD and fasted for 12 h before sacrifice. Jejunal segments were also isolated from mice fed chow diet, fasted for 12 h, and gavaged with 200 μl olive oil containing 1% cholesterol 2 h prior sacrifice. The tissues were fixed in 4% paraformaldehyde (Carl Roth, Karlsruhe, Germany) for 16 h at 4 °C and then transferred to 30% sucrose (Carl Roth) prior to cryosectioning. ORO staining was carried out as previously described [24].

### 2.10 RNA isolation and quantitative real-time PCR (qPCR)

Total RNA from tissues was isolated with TRI Reagent (Molecular Research Center, Inc., Cincinnati, OH). Two micrograms of RNA were reverse transcribed using the High-Capacity cDNA Reverse Transcription Kit (Applied Biosystems, Waltham, MA). qPCR was performed on a Bio-Rad CF X96 real-time PCR system (Bio-Rad Laboratories, Hercules, CA) using Luna® Universal qPCR Master Mix (New England Biolabs, Ipswich, MA). The samples were analyzed in duplicate, normalized to the expression of cyclophilin A (*Ppia*), and calculated using the 2^−ΔΔCT^ method. The primer sequences used are listed in Supplementary Table S1.

### 2.11 Isolation and loading of peritoneal macrophages with acetylated LDL

Mouse peritoneal macrophages (MPMs) were elicited by intraperitoneal injection of 3 % thioglycolate broth (50 μl/g body weight). Seventy-two hours post-injection, the cells were collected by flushing the peritoneum with ice-cold 1 mM EDTA in PBS. The cells were then centrifuged at 300×*g* for 5 min at 4 °C, after which they were resuspended in complete medium (DMEM containing high-glucose (4.5 g/l), 4 mM glutamine, 1 mM pyruvate, 10 % lipoprotein-deficient serum (LPDS), 1 % penicillin/streptomycin (P/S)), seeded in 6-well or 12-well plates, and adherent cells were washed 4 h later. After 24 h in culture, the cells were cultivated in complete medium for another 24 h in the absence or presence of 100 μg acetylated (ac)LDL protein/ml before harvesting.

### 2.12 Isolation of hepatocytes

Hepatocytes were isolated using a previously described protocol with minor modifications [25]. All buffers were pre-warmed to 37 °C. The livers were perfused with perfusion buffer (0.5 mM EDTA and 25 mM HEPES in Hanks' Balanced Salt Solution (HBSS) without Ca^2+^ and Mg^2+^) via the inferior vena cava, and blood was drained out from the portal vein until the liver appeared pale. The livers were then digested using 10 ml of digestion buffer (1 mg/ml Liberase™ (Sigma–Aldrich, St. Louis, MO), 25 mM HEPES in HBSS with Ca^2+^ and Mg^2+^), transferred to cold hepatocyte plating medium (DMEM low-glucose (1 g/l), 5% FBS, 1% P/S), and the liver cells were released by perforating the liver capsule and gently pressing the tissue. The obtained cell suspension was filtered through a 70-μm cell strainer and centrifuged at 50×*g* for 2 min at 4 °C. The pellet was resuspended in 10 ml plating medium, mixed with 10 ml of 90 % Percoll (Sigma–Aldrich) in PBS, and centrifuged at 200×*g* for 10 min at 4 °C. After washing of the pellet, the cells were seeded with plating medium in 10-cm dishes. Three hours later, the medium was changed to pre-warmed Williams E medium containing 1% l-glutamine and 1% P/S.

### 2.13 Isolation of enterocytes

Enterocytes were isolated from the duodenum, jejunum, and ileum as previously described with minor modifications [26]. Briefly, the intestine was opened up longitudinally and cleaned in PBS. The intestinal sections were washed in Buffer 1 (HBSS with 25 mM HEPES and 1% FBS), followed by washing in Buffer 2 (Ca^2+^- and Mg^2+^-free HBSS with 2% glucose and 2% BSA), after which the tissue was placed in Buffer 2 containing 1.5 mM EDTA. The intestinal segments were incubated for 15 min at 37 °C under rotation, briefly vortexed, and the supernatant containing the enterocytes was collected. The process was repeated and the supernatants containing the isolated enterocytes were combined. The cells were centrifuged for 5 min at 195×*g* and 25 °C and the cell pellets were washed again with Buffer 2.

### 2.14 Preparation of conditioned medium from macrophages and hepatocytes

Adherent MPMs from wild-type (WT) mice were cultivated in serum-free DMEM in the presence of 15 mM NH_4_Cl for 24 h to allow LAL secretion. Primary hepatocytes were cultivated in Williams E medium containing 1% l-glutamine and 1% P/S for 24 h. Thereafter, the media were collected and centrifuged at 3,113×*g* for 10 min at 4 °C. The supernatant was then transferred to Amicon® Ultracentrifuge filters (10 kDa MWCO; Merck Millipore, Darmstadt, Germany) and centrifuged at 3,113×*g* at 4 °C to concentrate to 150 times the original volume. The concentrated conditioned medium of MPMs or hepatocytes was added at a dilution of 1:20 to DMEM (containing high-glucose (4.5 g/l), 4 mM glutamine, 1 mM pyruvate, 20% FBS or 10% LPDS, 1 % P/S) on LAL KO MPMs. The medium was removed 36 h post-treatment, the cells were washed thrice with PBS and harvested for subsequent experiments.

### 2.15 Cholesterol quantification in MPMs

After washing the MPMs thoroughly with PBS, the lipids were extracted with 2 ml hexane:isopropanol (3:2, v:v) per well for 2 h at 4 °C under constant shaking. To the collected extracts, 100 μl of 2% Triton X-100 in chloroform was added and dried under a stream of nitrogen. The lipids were dissolved in 100 μl ddH_2_O for 30 min in an ultrasonic water bath. TC and FC concentrations were measured enzymatically using commercially available kits (DiaSys, Holzheim, Germany; mti Diagnostics GmbH, Idstein, Germany) and normalized to protein concentrations. The reagents were supplemented with sodium 3,5-dichloro-2-hydroxy-benzenesulfonate to enhance sensitivity. CE concentrations were calculated by subtracting FC from TC.

### 2.16 BODIPY staining of macrophages

MPMs were grown on glass coverslips for 24 h in complete DMEM containing 20% FBS. Thereafter, the cells were cultivated in the absence and presence of conditioned medium obtained from cultured WT MPMs for 24 h. The cells were washed with PBS, fixed with 4% paraformaldehyde (Carl Roth, Karlsruhe, Germany) for 10 min, and washed thrice with PBS-T (PBS containing 0.1% Triton X-100; Sigma–Aldrich, St. Louis, MO) for 5 min. Staining with BODIPY (1:200 in PBS; Thermo Fisher Scientific, Waltham, MA) was performed for 30 min. The cells were again washed with PBS-T and counterstained with DAPI (1:1,000 dilution in PBS; Thermo Fisher Scientific) for 3 min before being mounted with a fluorescent mounting medium (Agilent Technologies, Inc., Santa Clara, CA). The cells were imaged using an Olympus BX63 microscope equipped with an Olympus DP73 camera.

### 2.17 LAL activity assay

CE hydrolase activity at an acidic pH (pH ∼ 4.2), representing LAL activity, was measured as previously described with minor modifications [14]. Briefly, cell pellets were lysed in a lysis buffer (250 mM sucrose, 1 mM EDTA, 1 mM dithiothreitol, 1:2,000 protease inhibitor cocktail (PIC), pH 7.2) by sonication. Protein concentrations were determined using the DC™ Protein assay (Bio-Rad Laboratories, Hercules, CA). Thereafter, 100 μg of protein were diluted to a final volume of 100 μl in the lysis buffer. The substrate containing 0.2 mM cholesteryl oleate, 455 μM phosphatidylcholine:phosphatidylinositol (3:1), and 0.1 μCi of cholesteryl-[^14^C]-oleate (Amersham Biosciences, Piscataway, NJ) was dried under a stream of nitrogen and sonicated in 0.1 M citric acid buffer (pH 4.2) with 90% of the final substrate volume to form a lipid emulsion. Then, 2% fatty acid-free BSA (Biowest, Nuaillé, France) was added to the substrate. Substrate and sample (100 μl each) were mixed and incubated for 1 h at 37 °C in a shaking water bath. The reaction was terminated by the addition of 3.25 ml stop solution (methanol:choroform:heptane, 10:9:7, v:v:v) and 1 ml of 100 mM potassium carbonate, pH 10.5 adjusted with saturated boric acid (all Carl Roth, Karlsruhe, Germany). After vortexing and centrifugation at 2,400×*g* and 20 °C for 15 min, the radioactivity in 1 ml of the upper phase was determined by liquid scintillation counting, and the release of fatty acids was calculated as previously described [27].

### 2.18 Western blotting

Equal volumes of 1:10 diluted plasma samples or ratiometrically proportional volumes of conditioned medium were separated by SDS-PAGE and transferred to activated PVDF membranes, followed by incubation with an anti-rabbit LAL (PA5-27346, 1:1,000; Invitrogen, Waltham, MA) polyclonal antibody. Liver tissue lysates or plasma of LAL KO mice as well as purified recombinant mouse LAL served as controls. Secondary polyclonal goat anti-rabbit antibody (P0448, 1:1,000; Agilent Technologies, Inc., Santa Clara, CA) conjugated to HRP was visualized using the Clarity Western ECL Substrate (Bio-Rad Laboratories, Hercules, CA) on a ChemiDoc™ imaging system (Bio-Rad Laboratories).

### 2.19 Statistical analyses

Data are presented as mean ± SD. Statistical analyses were performed using GraphPad Prism software 10 (GraphPad Software Inc., San Diego, CA). Significance was calculated by unpaired Student's t-test for comparison of two groups and one-way or two-way ANOVA followed by Tukey *post-hoc* test for comparison of multiple groups. Significance levels were set at ∗p < 0.05, ∗∗p ≤ 0.01, ∗∗∗p ≤ 0.001, and ∗∗∗∗p ≤ 0.0001 and ^#^p < 0.05, ^##^p ≤ 0.01, ^###^p ≤ 0.001, and ^####^p ≤ 0.0001.

## 3 Results

### 3.1 Macrophage-specific LAL KO mice on chow diet display reduced hepatic lipid accumulation and circulating cholesterol concentrations

Mice with systemic LAL-D suffer from a drastic LAM infiltration in several organs, including the liver and small intestine, suggesting that these cells, in addition to hepatocytes, are responsible for the majority of ectopic lipid deposition [18]. To study the contribution of lysosomal lipid hydrolysis by macrophages to the LAL-D phenotype, we generated mice with specific loss of LAL in macrophages (macLAL-KO) and phenotypically characterized them under standard chow and after 12 weeks of feeding a HF/HCD (Suppl. Fig. 1A).

**Figure 1:**
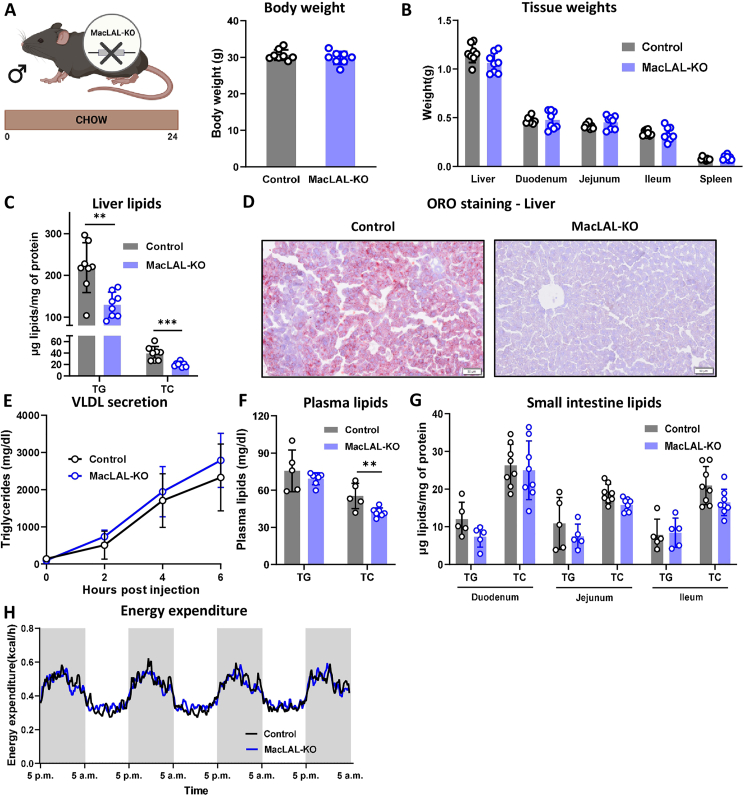
**Reduced hepatic lipid content in chow diet-fed macLAL-KO mice. (A)** Body and **(B)** tissue weights of male chow diet-fed macLAL-KO mice and controls measured at 25–27 weeks of age (n = 8). **(C)** Hepatic lipid parameters and **(D)** representative images of oil red O-stained liver sections (scale bar, 50 μm). **(E)** VLDL secretion of 12-week-old macLAL-KO and control mice (n = 5–7). **(F)** Plasma triglyceride (TG) and total cholesterol (TC) concentrations (n = 5–7). **(G)** Intestinal TG (n = 5) and TC content (n = 8). **(H)** Energy expenditure of macLAL-KO and control mice measured in metabolic cages (n = 5–7). Data are presented as mean values ± SD. ∗∗p ≤ 0.01, ∗∗∗p ≤ 0.001.

The absence of *Lipa* mRNA expression in bone marrow-derived macrophages (BMDMs) (Suppl. Fig. S1B) and peritoneal macrophages (MPMs) (Suppl. Fig. S1C) as well as the lack of CE hydrolase activity in BMDMs (Suppl. Fig. S1D) confirmed the successful KO. Chow diet-fed male macLAL-KO mice and their corresponding controls had comparable body (Figure 1A) and tissue weights (Figure 1B) at the age of 25–27 weeks. However, hepatic TG and TC content were significantly reduced in macLAL-KO mice compared to the controls (Figure 1C), which was confirmed by ORO staining of liver sections (Figure 1D). Unchanged VLDL secretion from the liver (Figure 1E) was consistent with comparable plasma TG concentrations between the genotypes, whereas cholesterol levels were decreased in macLAL-KO mice (Figure 1F). In contrast, TG and TC content in the three segments of the small intestine (duodenum, jejunum, ileum) were unaltered (Figure 1G). When monitored in metabolic cages, macLAL-KO mice and their corresponding controls had comparable energy expenditure (Figure 1H), water intake (Suppl. Fig. S1E), and food consumption (Suppl. Fig. S1F). From these findings we concluded that chow diet-fed macLAL-KO mice failed to replicate the phenotype observed in systemic LAL KO mice.

### 3.2 Macrophage-specific LAL KO mice are protected from diet-induced obesity

**Figure 2:**
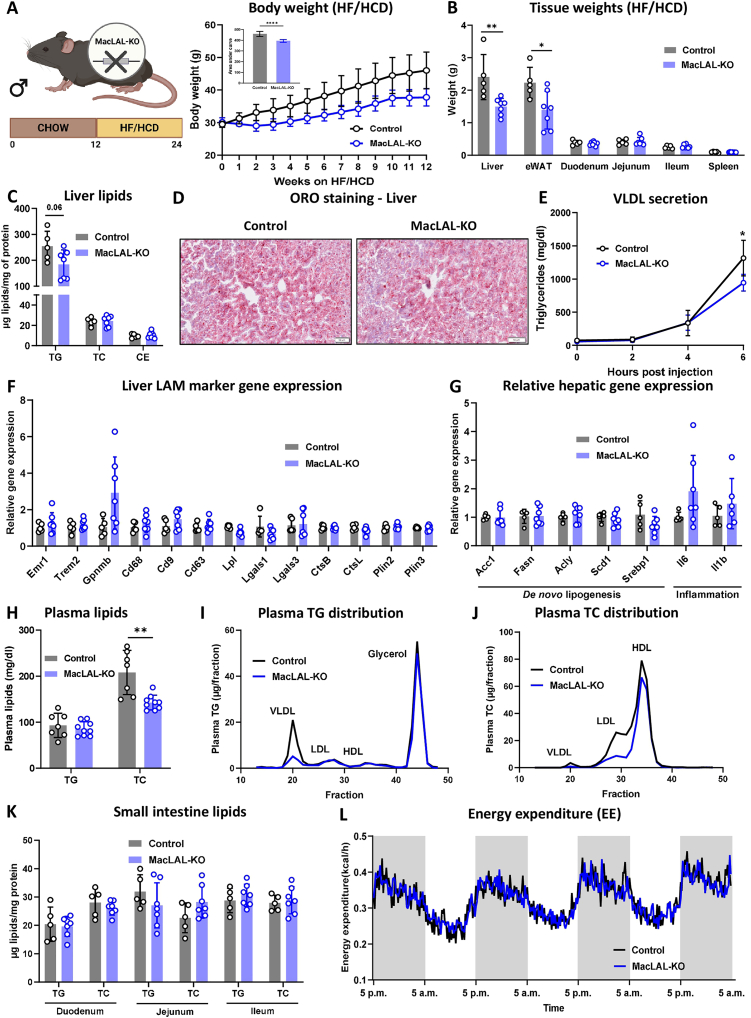
**MacLAL-KO mice are protected from diet-induced obesity. (A)** Body weights during and **(B)** tissue weights after 12-week HF/HCD feeding of male macLAL-KO and control mice (n = 5–7). **(C)** Hepatic lipid parameters, **(D)** representative images of oil red O-stained liver sections (scale bar, 50 μm), and **(E)** VLDL secretion in HF/HCD-fed mice (n = 5–7). Relative gene expression of **(F)** LAM marker genes and **(G)** genes involved in *de novo* lipogenesis and inflammation in the liver measured by qPCR (n = 5–7). **(H)** Plasma triglyceride (TG) and total cholesterol (TC) concentrations (n = 7–9) and lipoprotein profiles of **(I)** TG and **(J)** cholesterol in pooled plasma samples (n = 7–9) after separation by fast protein liquid chromatography. **(K)** TG and TC concentrations of control and macLAL-KO small intestines (n = 5–7). **(L)** Energy expenditure of macLAL-KO and control mice measured in metabolic cages (n = 5–7). Data are presented as mean values ± SD. ∗p ≤ 0.05, ∗∗p ≤ 0.01, ∗∗∗∗p ≤ 0.0001.

Next, we challenged macLAL-KO mice with a HF/HCD for 12 weeks to induce a high lipid load. MacLAL-KO mice had an attenuated diet-induced weight gain (Figure 2A) that was reflected by reduced liver and epididymal (e)WAT weights, whereas the three segments of the small intestine and the spleen remained comparable between the genotypes (Fig. 2B). Surprisingly and in contrast to chow diet-fed macLAL-KO mice, we detected an unchanged cholesterol content and only a mild reduction in hepatic TG in HF/HCD-fed macLAL-KO mice (Figure 2C), in line with ORO staining (Figure 2D) and only slightly altered VLDL-TG secretion (Figure 2E). Comparable expression levels of genes associated with LAMs indicated the absence of LAM accumulation in the livers of macLAL-KO (Fig. 2F). Moreover, genes linked to *de novo* lipogenesis and hepatic inflammation remained unaffected (Fig. 2G). In line with chow diet-fed mice, circulating cholesterol levels were significantly reduced in HF/HCD-fed macLAL-KO animals (Figure 2H). Despite unaltered circulating TG levels, lipoprotein profiling after FPLC separation revealed reduced TG concentrations in the VLDL fraction (Figure 2I) and markedly lower TC content in the LDL and HDL fractions of macLAL-KO plasma (Figure 2J). However, intestinal lipid levels again remained unaltered between the genotypes (Figure 2K). In addition, energy expenditure (Figure 2L) as well as water and food intake were unchanged (Suppl. Fig. 1G and H). Overall, the loss of LAL in macrophages partially protected the mice from developing diet-induced obesity and hypercholesterolemia. However, the adverse phenotype observed in whole-body LAL KO mice could not be reproduced in macLAL-KO mice, even when fed a HF/HCD.

### 3.3 Macrophage/enterocyte-specific LAL KO mice do not reflect the pathologic phenotype of global LAL KO mice

To delineate the role of epithelial versus subepithelial cells in the pathologic malabsorption in LAL-D, we crossed macLAL-KO with enterocyte-specific LAL-KO mice [18] to generate KO mice with a combined deletion of LAL expression in macrophages and enterocytes. In agreement with the lack of *Lipa* mRNA expression in enterocytes (Suppl. Fig. S2A), CE hydrolase activity in duodenum, jejunum, and ileum was comparable between mac/int-LAL KO and whole-body LAL KO mice (Suppl. Fig. S2B).

**Figure 3:**
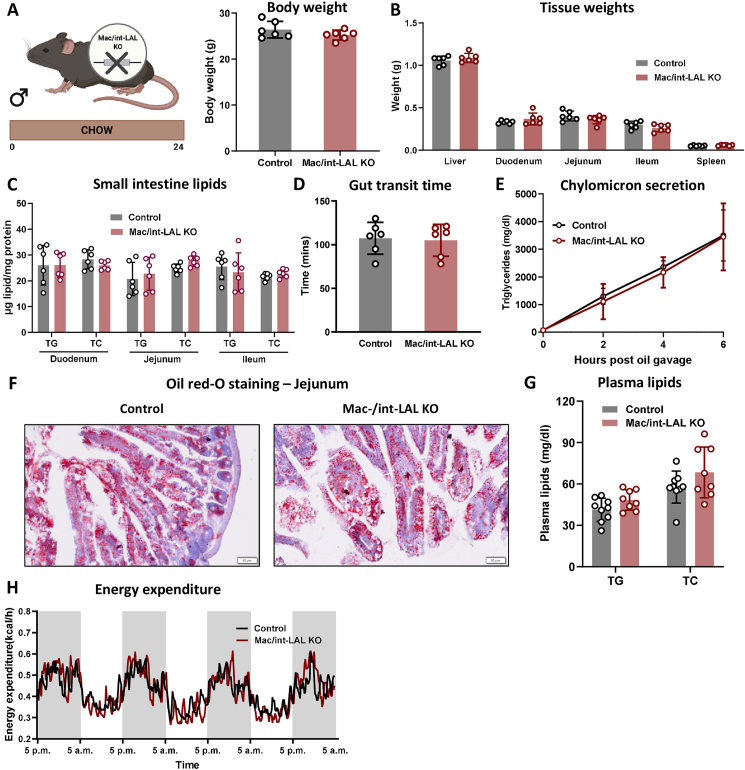
**Mac/int-LAL KO mice show no obvious phenotype on chow diet. (A)** Body and **(B)** tissue weights of male chow diet-fed mac/int-LAL KO and control mice sacrificed at 24–27 weeks of age (n = 6). **(C)** Triglyceride (TG) and total cholesterol (TC) concentrations in the three segments of the small intestine (n = 6). **(D)** Gut transit time (n = 6) and **(E)** chylomicron secretion in mac/int-LAL KO and control mice (n = 5–7; 1 significant outlier excluded). **(F)** Representative images of oil red O-stained sections from the jejunum post gavage of olive oil plus 1% cholesterol (scale bar, 100 μm). **(G)** Plasma TG and TC concentrations (n = 8–9; 1 significant outlier per group excluded). **(H)** Energy expenditure of mac/int-LAL KO and control mice measured in metabolic cages (n = 3). Data are presented as mean values ± SD.

In line with macLAL KO and intLAL KO mice [18], male 24-week-old chow diet-fed mac/int-LAL KO mice had similar body (Figure 3A) and tissue weights (Fig. 3B) as the corresponding controls. We failed to detect any differences in intestinal TG and cholesterol levels (Fig. 3C), gut transit time (Fig. 3D), and chylomicron secretion upon oral oil administration (Fig. 3E), indicating unaltered intestinal absorption in mac/int-LAL KO mice. ORO staining of jejunal sections (Fig. 3F) post-gavage with olive oil containing 1% cholesterol indicated comparable lipid distribution between the two groups despite an acute high-fat, high-cholesterol challenge. Plasma lipid parameters (Fig. 3G), energy expenditure (Fig. 3H), as well as water and food intake (Suppl. Fig. S2C and D) were comparable between mac/int-LAL KO and control mice. Taken together, the simultaneous loss of LAL in macrophages and enterocytes in mice fed chow diet failed to reproduce the lipid-filled small intestinal phenotype observed in whole-body LAL KO mice.

**Figure 4:**
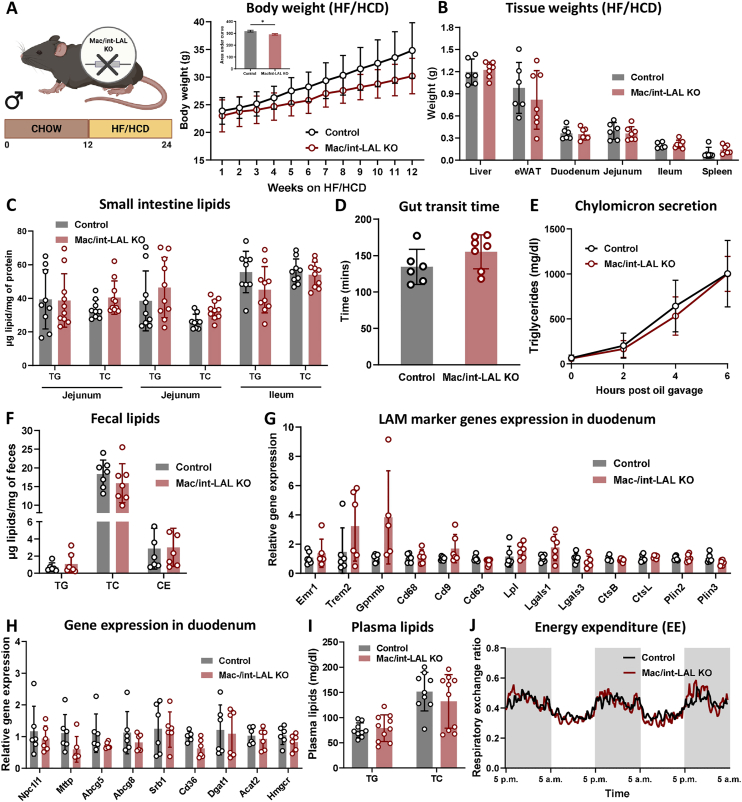
**Mac/int-LAL KO mice remain unaffected by HF/HCD feeding. (A)** Body weights during and **(B)** tissue weights after 12-week HF/HCD feeding of male mac/int-LAL KO and control mice (n = 9–10). **(C)** Tissue lipid parameters in the three segments of the small intestine (n = 9–10). **(D)** Gut transit time (n = 6–7), **(E)** chylomicron secretion (n = 8–9; 1 significant outlier per group excluded), and **(F)** fecal lipid estimations (n = 6–7). Relative mRNA expression levels of **(G)** LAM markers and **(H)** genes associated with lipid uptake and transport in the duodenum of control and mac/int-LAL KO mice measured by qPCR (n = 6). **(I)** Plasma TG and TC concentrations (n = 9–10) upon HF/HCD feeding. **(J)** Energy expenditure measured in metabolic cages (n = 3–4). Data are presented as mean values ± SD. ∗p ≤ 0.05.

To challenge intestinal lipid metabolism, mac/int-LAL KO mice were fed a HF/HCD for 12 weeks. Consistent with intLAL-KO [18] and macLAL-KO mice (Fig. 2), body weight gain was attenuated in mac/int-LAL KO mice compared to their controls (Figure 4A). This reduction, however, was less pronounced than in macLAL KO mice, likely due to the decreased weight gain of the control animals. Tissue weights of liver, eWAT, spleen, and intestine were comparable between the genotypes (Fig. 4B). Additionally, intestinal lipid concentrations upon 12 h of fasting remained unaltered (Fig. 4C), as further confirmed by ORO staining of sections from duodenum, jejunum, and ileum (Suppl. Fig. S3A–C). Similarly, gut transit time (Fig. 4D), chylomicron secretion (Fig. 4E), and fecal lipid excretion (Fig. 4F) were also unchanged, suggesting that even the simultaneous loss of LAL in both enterocytes and macrophages failed to affect intestinal lipid homeostasis. Unaltered mRNA expression of LAM marker genes in the duodenum (Fig. 4G), jejunum (Suppl. Fig. S3D), and ileum (Suppl. Fig. S3E) indicated an absence of infiltrating monocyte-derived macrophages that are present in systemic LAL KO mice. Expression of lipid transport genes in the intestine were also comparable between the genotypes in all three parts of the small intestine (Figure 4H, Suppl. Fig. S3F and G). Along with unchanged plasma lipid levels (Fig. 4I), energy expenditure (Fig. 4J), water and food intake (Suppl. Fig. S3H and I), these results demonstrated the absence of a typical intestinal LAL-D phenotype in mac/int-LAL KO mice, even when fed a HF/HCD.

### 3.4 MacLAL-KO macrophages take up functional LAL secreted from control macrophages and hepatocytes

**Figure 5:**
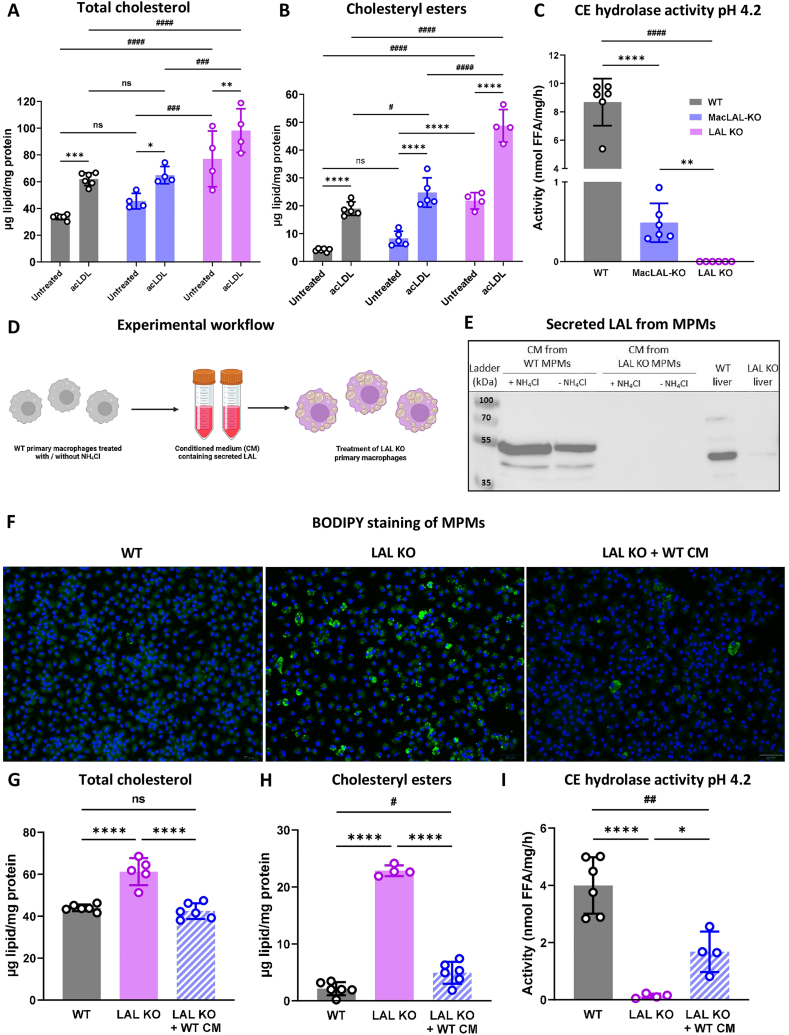
**Uptake of secreted LAL corrects the phenotype in LAL KO macrophages. (A)** Total cholesterol and **(B)** CE accumulation in wild-type (WT), macLAL-KO, and LAL KO mouse peritoneal macrophages (MPMs) in the absence and presence of acetylated LDL (n = 4–6). **(C)** CE hydrolase activity at acidic pH in MPMs 24 h post seeding (n = 6). **(D)** Experimental workflow for the preparation of conditioned medium (CM) from WT MPMs. **(E)** Western blot of LAL in CM collected from WT MPMs cultivated in the absence or presence of NH_4_Cl. **(F)** BODIPY staining in WT and LAL KO MPMs with or without WT CM treatment (scale bar, 50 μm). **(G)** Total cholesterol and **(H)** CE concentrations as well as **(I)** CE hydrolase activity (pH 4.2) in MPMs from LAL KO mice cultivated in the absence or presence of WT CM (n = 4–6). Data are presented as mean values ± SD. ∗p ≤ 0.05, ∗∗p ≤ 0.01, ∗∗∗p ≤ 0.001, ∗∗∗∗p ≤ 0.0001, ^#^p ≤ 0.05, ^##^p ≤ 0.01, ^###^p ≤ 0.001, ^####^p ≤ 0.0001.

The results obtained so far suggested that the release of LAL from LAL-expressing cells (e.g. hepatocytes) may correct the phenotype that would have been caused by the genetic loss of the enzyme. Consistent with this notion, MPMs isolated from macLAL-KO mice lacked the tremendous cholesterol and CE accumulation observed in LAL-KO MPMs, even after loading the cells with acLDL (Figure 5A–B). In addition, the residual acidic CE hydrolase activity in MPMs from macLAL-KO mice that is absent in global LAL KO MPMs (Fig. 5C) indicated that macLAL-KO MPMs have taken up functional LAL from other cells.

To investigate whether macrophages are capable of secreting (functional) LAL, we collected conditioned medium from WT MPMs and incubated LAL-KO MPMs with it (Fig. 5D). Of note, NH_4_Cl treatment was used to disrupt proper sorting and trigger the secretion of lysosomal enzymes [28], resulting in increased LAL secretion (Fig. 5E). BODIPY staining revealed that the foamy phenotype of LAL KO macrophages was absent after the treatment of LAL-KO MPMs with the conditioned medium from WT MPMs (Fig. 5F). This finding was confirmed by the reduction in cholesterol levels, particularly the CE accumulation in LAL KO macrophages reaching WT levels (Figure 5G,H). This simultaneously corresponded to a partial restoration of CE hydrolase activity in LAL KO MPMs following treatment (Fig. 5I).

**Figure 6:**
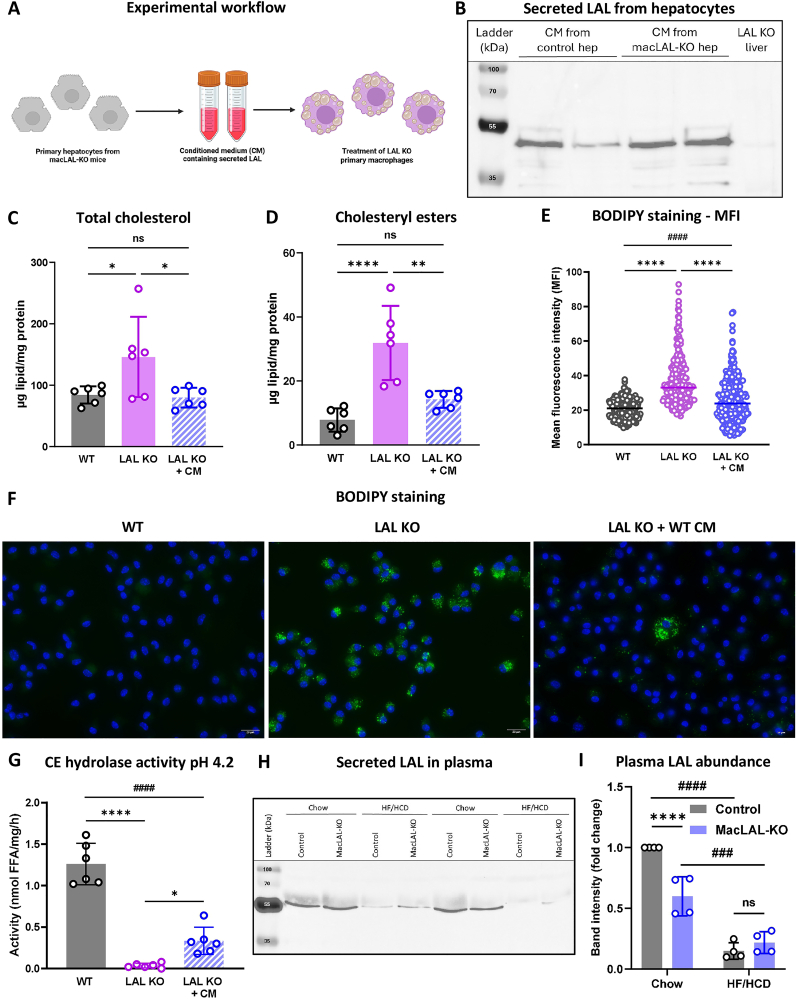
**Uptake of LAL secreted from hepatocytes corrects the phenotype of macLAL-KO macrophages. (A)** Experimental workflow for the preparation of conditioned medium (CM) from macLAL-KO hepatocytes. **(B)** LAL protein abundance in CM collected from macLAL-KO primary hepatocytes 24 h post-seeding. **(C)** Total cholesterol and **(D)** CE concentrations in LAL KO MPMs cultivated in the absence or for 36 h with WT CM, compared to WT cells (n = 6). **(E)** Quantification of mean fluorescence intensity and **(F)** representative images of BODIPY staining in WT and LAL KO MPMs with and without CM treatment (scale bar, 20 μm). **(G)** CE hydrolase activity (pH 4.2) in LAL KO MPMs with and without CM treatment (n = 4). **(H)** Representative Western blot showing LAL protein abundance in the plasma of macLAL-KO and control mice fed chow or 12-weeks HF/HCD. (**I**) Quantification of circulating LAL abundance as fold change (n = 4). Data are presented as mean values ± SD. ∗p ≤ 0.05, ∗∗p ≤ 0.01, ∗∗∗∗p ≤ 0.0001, ^###^p ≤ 0.0001, ^####^p ≤ 0.0001.

To elucidate the potential compensatory mechanism that may occur *in vivo*, we tested whether primary hepatocytes could correct LAL-D by acting as enzyme donors using a similar experiment (Figure 6A), since they express high levels of LAL and have also been implicated in LAL-D pathology [29]. Primary hepatocytes isolated from control and macLAL-KO mice secreted comparable amounts of LAL (Fig. 6B). Moreover, conditioned medium collected from macLAL-KO hepatocytes reduced cholesterol and CE accumulation in LAL KO MPMs comparable to conditioned medium from WT MPMs (Figure 6C,D). Consistently, the foamy appearance normally seen in LAL KO macrophages was absent after the treatment, as visualized by BODIPY staining (Figure 6E,F). Enzyme activity (i.e., acidic CE hydrolase activity) in LAL KO macrophages was also partially restored (Fig. 6G). LAL protein expression was detected in the plasma of macLAL-KO mice (Fig. 6H), further confirming that cells other than macrophages can secrete LAL into the circulation. Of note, the abundance of LAL in the plasma was dependent on the diet, revealing a significant decrease in circulating LAL after 12-weeks of HF/HCD feeding in macLAL-KO and control mice (Figure 6H,I). Overall, these results indicate that functional LAL secreted into the circulation by LAL-expressing cells can compensate for the phenotype in cell-specific LAL KO mouse models.

## 4 Discussion

LAL-D is a rare genetic disorder characterized by severely impaired lipid metabolism, particularly affecting the liver and small intestine. In agreement with LAL-D patients, LAL KO mice exhibit a drastic dysregulation of lipid biogenesis and absorption, with ectopic lipid accumulation in multiple organs [12]. Hepatocyte-specific (hep)LAL-KO mice only partially replicated the liver phenotype of LAL-D, and only after HF/HCD feeding [30]. Moreover, enterocyte-specific LAL KO mice had no detectable phenotype on either chow or HF/HCD, which strongly contrasts the phenotypic changes in intestinal absorption observed in LAL KO mice [13,18]. However, both the liver and the small intestine of LAL KO mice show a massive infiltration of foamy macrophages and the accumulation of CE crystals, indicating that these cells are among the main drivers of the ectopic lipid accumulation in LAL-D. Consistent with this, macrophages infiltrating the lamina propria of LAL KO mice are CD68^+^TREM2^+^GPNMB^+^ cells, referred to as LAMs [18]. LAMs have been extensively studied in several metabolic diseases such as obesity, metabolic dysfunction-associated steatotic liver disease, and atherosclerosis, where they colonize the WAT, liver, and the intima of the arterial wall, respectively [[31], [32], [33], [34], [35], [36], [37]]. Under these pathological conditions, LAMs interact with lipoproteins, clear apoptotic cells, and participate in the remodeling of the extracellular matrix to initially sequester and alleviate lipid overload in the tissue [38]. The role of LAMs as pro- or anti-inflammatory mediators remains controversial and varies by disease context (summarized in [31,39]). Their function in LAL-D remains undefined.

To evaluate the role of macrophages in the pathology of LAL-D and to assess their contribution to the metabolic dysfunction, lipid uptake, and inflammation in the affected organs, we generated and characterized mac- and mac/int-specific LAL KO mice. Despite reduced hepatic steatosis, resistance to diet-induced obesity, lower liver and epididymal WAT mass, and systemically decreased circulating cholesterol levels in macLAL-KO mice, the lipid content and infiltration of lipid-filled macrophages in the liver were unaltered. Mac/int-LAL KO were similarly protected from diet-induced obesity, but intestinal lipid absorption remained unchanged. We infer that the phenotype is attributable to macrophage LAL-D and might be explained by altered lipid handling and partitioning, manifested as reduced VLDL output and diminished adipose lipid deposition, rather than by a large, quantifiable shift in the primary components of energy balance. However, our data do not mechanistically resolve the energy balance discrepancy. One study limitation is that the two control cohorts differed in body and tissue weight gain (with macLAL-KO controls being heavier than mac/int-LAL KO controls), potentially amplifying macLAL-KO effects. Mice lacking LAL specifically in hepatocytes are also resistant to diet-induced obesity with markedly decreased WAT mass that might be due to an increased energy demand [30]. Dietary stress by HF/HCD provokes the formation of fatty lysosomes and CE crystals in hepatocytes of hepLAL-KO mice, accompanied by liver inflammation, inflammasome activation, and triggering of a macrophage “repair” response, leading to their infiltration into the tissue and further exacerbation of damage [30]. Notably, lipid accumulation in the small intestine of whole-body LAL KO mice arises from systemic inflammation and circulating immune cell infiltration [13], which are absent in enterocyte-specific [18] and macLAL-KO mice.

The absence of fatty lysosomes in the villi of mac/int-LAL KO mice was puzzling. Prior studies with primary human fibroblasts have shown that LAL can be secreted into the extracellular space by fibroblasts [40,41] and be taken up by mannose-6-phosphate receptor-mediated endocytosis [42]. Studies in LAL KO mice with restored LAL expression in hepatocytes [21,29,43] or macrophages [44] and in LAL-D patients with hematopoietic re-introduction of LAL [45,46] revealed ameliorated pathogenesis of the disease, implying that secreted LAL is taken up by LAL-deficient cells. Consequently, this uptake mechanism established the basis for the development of an enzyme replacement therapy for LAL-D [[47], [48], [49], [50], [51]]. However, incomplete correction in hepLAL-KO mice [30] and persistent phenotype in adipocyte- and endothelial cell-specific LAL KO mice [52,53] indicate cell type-specific limits to enzyme uptake.

Consistent with cross-correction, MPMs isolated from macLAL-KO mice retained ∼5.5% LAL activity despite genetic *Lipa* deletion. This remaining LAL activity is apparently sufficient to prevent CE accumulation in lysosomes as commonly observed in LAL KO macrophages. Exposure to conditioned media from macrophages and hepatocytes demonstrated that both cell types are capable of secreting functional LAL, leading to correction of the CE accumulation in LAL KO macrophages back to WT levels. This was accompanied by a significant increase in enzyme activity, reaching 17% of the activity levels observed in WT macrophages. Notably, human patients with more than 10% residual LAL activity are considered to be not affected by LAL-D [54]. *In vivo*, residual LAL activity was detected only in MPMs, but not in *in vitro* differentiated BMDMs, confirming both the knockout validity and the requirement for cellular exposure to external enzyme for phenotype correction. Detectable LAL in macLAL-KO plasma further supports systemic cross-correction. Nevertheless, this rescue did not abrogate the protection from HF/HCD-induced weight gain in either macLAL-KO and mac/int-LAL-KO mice. We attribute this to dietary lipid overload that likely exacerbates lysosomal burden in macrophages and causes a supply/demand mismatch, whereby macrophage substrate load rises with dietary lipid excess, while the availability and/or delivery efficiency of hepatocyte-derived LAL for cross-correction declines. Notably and unlike the intestinal tissue, HF/HCD increases the relative abundance of adipose tissue macrophages, thereby potentiating adipocyte-macrophage crosstalk under pathological conditions. In contrast, lack of substantial infiltration of the lamina propria by circulating monocytes prevents the intestinal phenotype from worsening in mac/int-LAL DKO animals. Taken together, cross-correction that suffices on chow may become less effective under HF/HCD.

## 5 Conclusion

Collectively, our results provide evidence that cell type-specific LAL KO mouse models, particularly mac-, int-, and mac/int-specific LAL-KO mice, are unable to replicate the phenotype of global LAL KO mice, likely due to intercellular cross-correction via LAL secretion and uptake. Along with previous observations, we consider multiple cell types being involved in the pathogenesis of LAL-D. Our findings indicate that macrophages and hepatocytes are major sources of circulating LAL and that macrophages are also capable of LAL uptake, which will be critical for optimizing gene and enzyme-replacement therapies. Key uncertainties remain to be clarified regarding which cell types predominantly donate versus receive LAL under different dietary or metabolic conditions. Future studies should uncover the molecular and signaling mechanisms that drive macrophage infiltration and the subsequent ectopic lipid accumulation, chronic inflammation, and metabolic dysfunction to enable more targeted therapeutic strategies for LAL-D.

## CRediT authorship contribution statement

**Suravi Mukherjee:** Writing – review & editing, Writing – original draft, Methodology, Investigation, Formal analysis, Conceptualization. **Melanie Korbelius:** Writing – review & editing, Methodology, Investigation. **Anita Pirchheim:** Writing – review & editing, Methodology, Investigation. **Birgit Schwarz:** Writing – review & editing, Methodology, Investigation. **Malena Diaz:** Writing – review & editing, Methodology, Investigation. **Laszlo Schooltink:** Writing – review & editing, Methodology, Investigation. **Gernot F. Grabner:** Writing – review & editing, Resources, Methodology, Funding acquisition. **Nemanja Vujić:** Writing – review & editing, Supervision, Methodology, Investigation, Formal analysis, Conceptualization. **Dagmar Kratky:** Writing – review & editing, Supervision, Resources, Methodology, Funding acquisition, Conceptualization.

## Declaration of generative AI and AI-assisted technologies in the manuscript preparation process

During the preparation of this work, the authors used DeepL and Copilot to enhance clarity and improve the quality of the English language. After using this tool/service, the author(s) reviewed and edited the content as needed and take full responsibility for the content of the published article.

## Funding

This work was supported by the Austrian Science Fund (10.13039/501100002428FWF) (SFB 10.55776/F73, 10.55776/P32400, and DK-MCD 10.55776/W1226 awarded to D.K., 10.55776/AST7750924 awarded to G.F.G.), the flagship project “VascHealth” and the Ph.D. program “Molecular Medicine” of the 10.13039/501100010109Medical University of Graz, the 10.13039/501100009818Province of Styria, and the City of Graz. For open access purposes, the authors have applied a CC BY public copyright license to any author accepted manuscript version arising from this submission.

## Declaration of competing interest

The authors declare that no conflict of interest exists.

## Data Availability

Data will be made available on request.

## Footnotes

Appendix A Supplementary data to this article can be found online at https://doi.org/10.1016/j.molmet.2026.102369.
