# Supplementary material for: Secreted enzyme uptake masks the in vivo phenotype of macrophage-specific lysosomal acid lipase deletion
Source: Mol Metab. 2026 Apr 16;108:102369. doi: 10.1016/j.molmet.2026.102369 (PMC13138220; doi:10.1016/j.molmet.2026.102369)
Supplement: Multimedia component 1 [file mmc1.pdf]

Supplementary information

Secreted enzyme uptake masks the in vivo phenotype of macrophage-specific lysosomal acid lipase deletion

Suravi Mukherjee, Melanie Korbelius, Anita Pirchheim, Birgit Schwarz, Malena Diaz, Laszlo Schooltink, Gernot F. Grabner, Nemanja Vujić, and Dagmar Kratky

Supplementary Figures S1-S3

Supplementary Table S1

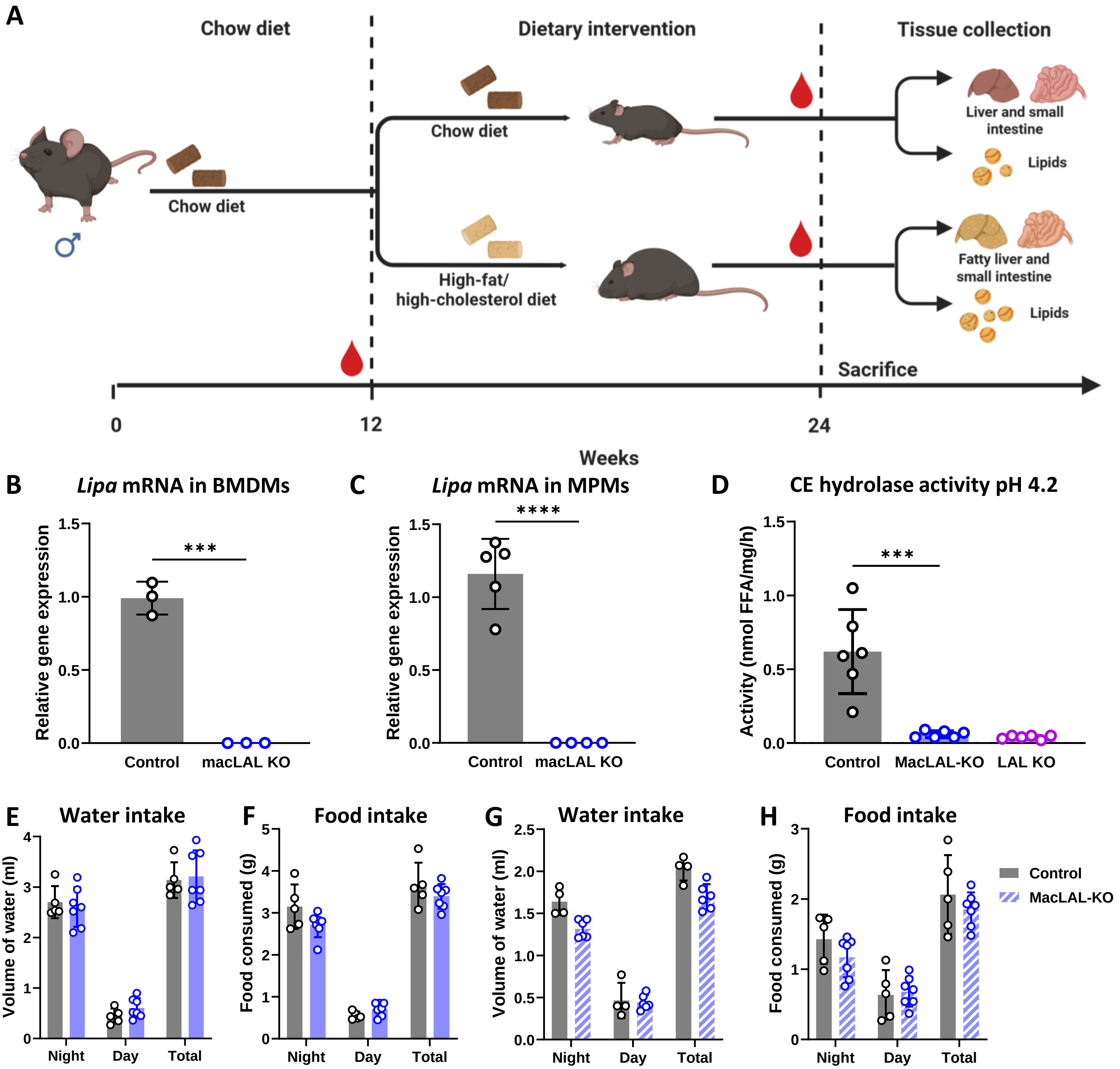

Supplementary Figure S1. (A) Detailed experimental workflow for characterizing all mouse cohorts with different feeding strategies. Expression of *Lipa* mRNA in macLAL-KO **(B)** bone marrow-derived macrophages (BMDMs) and **(C)** mouse peritoneal macrophages (MPMs) compared to their corresponding controls. **(D)** Cholesteryl ester (CE) hydrolase activity (pH 4.2) in BMDMs from macLAL-KO mice compared to control and systemic LAL KO mice (n = 6). Water and food intake from macLAL-KO mice and corresponding controls fed with **(E, F)** chow diet or **(G, H)** HF/HCD measured in metabolic cages (n = 5-7). Data are presented as mean values ± SD. ***p ≤ 0.001, ****p ≤ 0.0001.

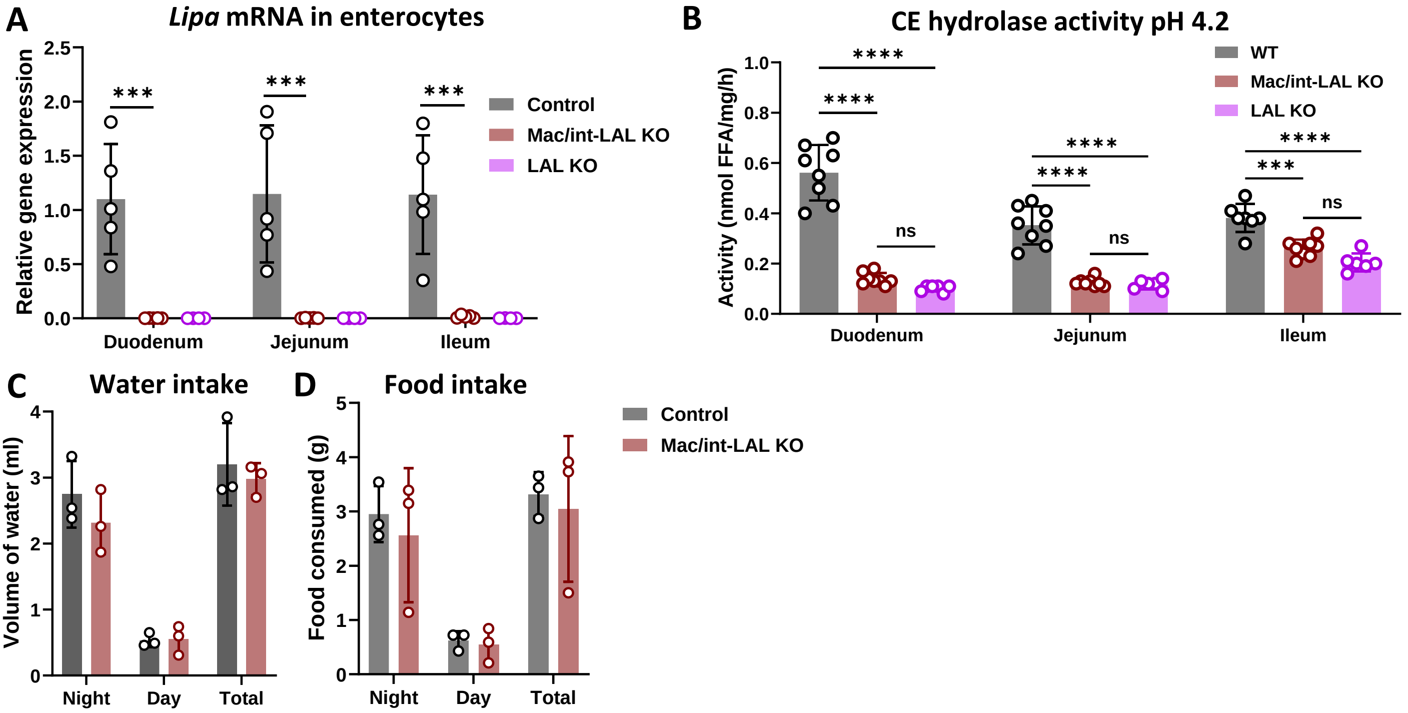

Supplementary Figure S2. (A) Expression of *Lipa* mRNA measured by qPCR and **(B)** cholesteryl ester (CE) hydrolase activity (pH 4.2) in isolated enterocytes from 3 segments of the small intestine from mac/int-LAL KO mice compared to control and global LAL KO mice (n = 6-8). **(C)** Water and **(D)** food intake measured in metabolic cages (n = 3). Data are presented as mean values ± SD. ***p ≤ 0.001, ****p ≤ 0.0001.

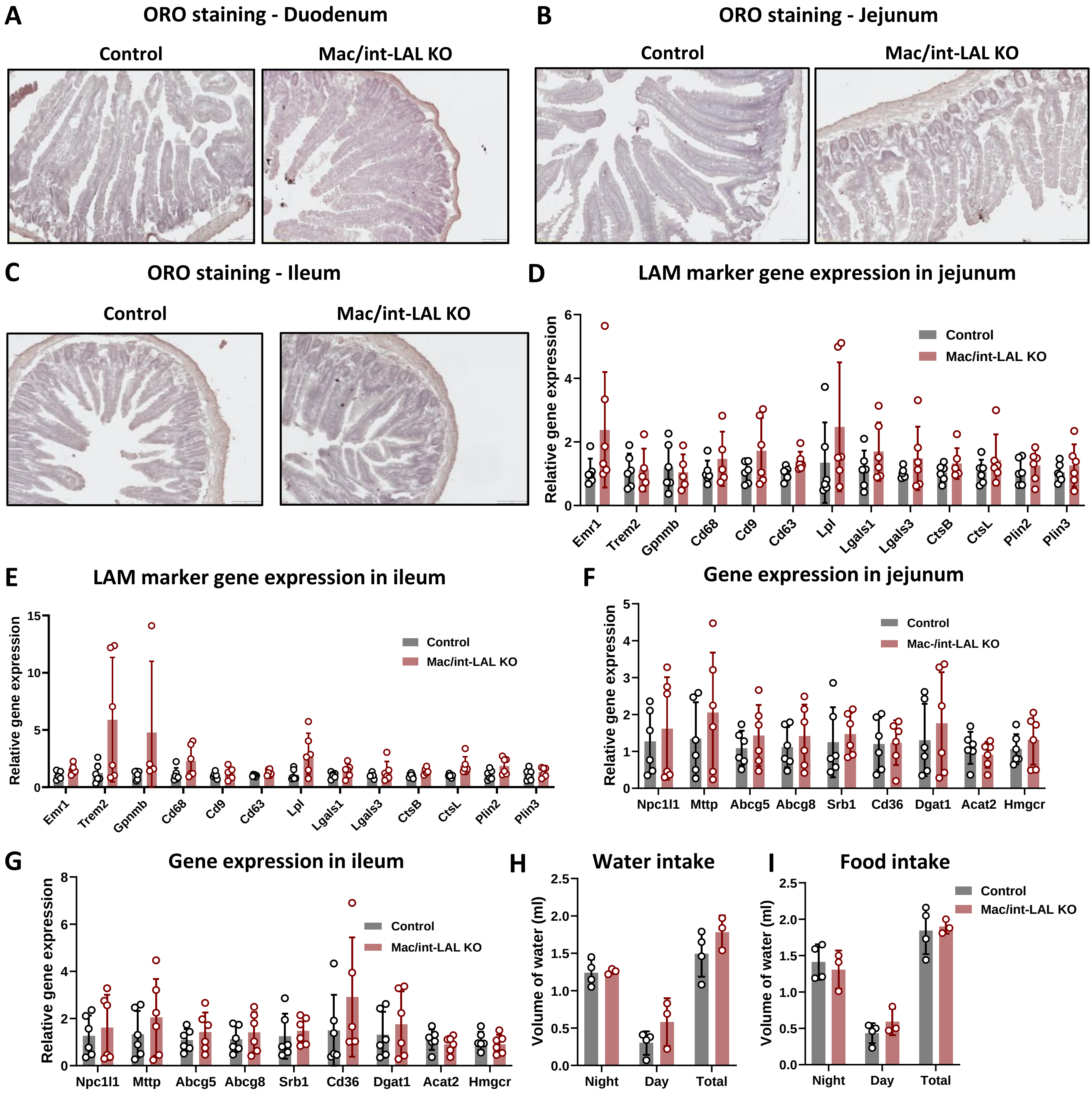

Supplementary Figure S3. Lack of small intestinal phenotype upon HF/HCD feeding in mac/int-LAL KO mice. Representative oil red O (ORO)-stained images of sections from control and mac/int-LAL KO **(A)** duodenum, **(B)** jejunum, and **(C)** ileum upon HF/HCD feeding for 12 weeks and 12-h fasting prior to sacrifice (scale bar, 100 µm). LAM marker gene expression in **(D)** jejunum and **(E)** ileum of mac/int-LAL KO mice and corresponding controls post HF/HCD feeding (n = 6). Expression of genes involved in lipid absorption and transport in **(F)** jejunum and **(G)** ileum (n = 6). **(H)** Water and **(I)** food intake measured in metabolic cages (n = 3-4). Data are represented as mean values ± SD.

Supplementary Table S1. Primer sequences used for real-time qPCR.

Gene	Forward primer	Reverse primer
Abcg5	AGAGGGCCTCACATCAACAGA	CTGACGCTGTAGGACACATGC
Abcg8	CTGTGGAATGGGACTGTACTTC	GTTGGACTGACCACTGTAGGT
Acat2	GATGGTCTGACAGATGCCTT	AGCACTGGCACAATCTCCTT
Acc1	GGACTTGGAGCAGAGAACCTTCG	CAAGCTGGTTGTTGGAGGTGTA
Acly	ACCCTTTCACTGGGGATCACA	GACAGGGATCAGGATTTCCTTG
Cd36	GCAGGTCTATCTACGCTGTG	GGTTGTCTGGATTCTGGAGG
Cd63	AGAGACCAGGTGAAGTCAGAG	AGTCTGTGTAGTTAGAAGCTCCA
Cd68	AACAGGACCTACATCAGAGC	TCAAGGTGAACAGCTGGAGA
Cd9	TGGGATTGTTCTTCGGGTTCC	TCCTTGTGGGTATAGCCCCAG
CtsB	TTGCGTTCGGTGAGGACATAG	GCAGGAGCCCTGGTCTCTA
CtsL	ACAGAAGACTGTATGGCACGA	GTATTCCCCGTTGTGTAGCTG
Dgat1	GTGCCATCGTCTGCAAGATTC	GCATCACCACACACCAATTCAG
Emr1	CTTTGGCTATGGGCTTCCAGTC	GCAAGGAGGACAGAGTTTATCGTG
Fasn	GAAGCCGAACACCTCTGTGCAGT	GCTCCTTGCTGCCATCTGTATTG
Gpnmb	GCTGGTCTTCGGATGAAAATGA	CCACAAAGGTGATATTGGAACCC
Hmgcr	TGTTCACCGGCAACAACAAGA	CCGCGTTATCGTCAGGATGA
Il1b	GAAATGCCACCTTTTGACAGTG	TGGATGCTCTCATCAGGACAG
Il6	CTGCAAGAGACTTCCATCCAG	AGTGGTATAGACAGGTCTGTTGG
Lgals1	CAAGCTGCCAGACGGACAT	AGGCCACGCACTTAATCTTGA
Lgals3	GGAGAGGGAATGATGTTGCCT	TCCTGCTTCGTGTTACACACA
Lipa	GCTGGCTTTGATGTGTGGATG	ATGGTGCAGCCTTGAGAATGA
Lpl	AGGACCCCTGAAGACACAG	ACATTCCCGTTACCGTCCATC
Mttp	GTCAACAGAGAGGCGAGAAG	CTAGCCAAGCCTCTCTTGAG
Npc1l1	TGTCCCCGCCTTATACAATGG	CCTTGGTGATAGACAGGCTACTG
Plin2	CTTGTGTCCTCCGCTTATGTC	GCAGAGGTCACGGTCTTCAC
Plin3	ATGTCTAGCAATGGTACAGATGC	CGTGGAACTGATAAGAGGCAGG
Scd1	GCTCTGGGGATGACTCTGAC	AGCCACACGTCCTTCCAAGTG
Srb1	TTTGGAGTGGTAGTAAAAAGGGC	TGACATCAGGGACTCAGAGTAG
Srebp1	CACTCAGCAGCCACCATCTAGCCT	GCTGATGCCTGCAGTCTTCACG
Trem2	CTGGAACCGTCACCATCACTC	CGAAACTCGATGACTCCTCGG
